# Effect of BNP on risk assessment in cardiac surgery patients, in addition to EuroScore II

**DOI:** 10.1038/s41598-020-67607-0

**Published:** 2020-07-02

**Authors:** Gaspard Suc, Philippe Estagnasie, Alain Brusset, Niki Procopi, Pierre Squara, Lee S. Nguyen

**Affiliations:** Centre Médico-Chirurgical Ambroise Paré, Critical Care Medicine Department, Recherche & Innovation de La Clinique Ambroise Paré (RICAP), 25 Boulevard Victor Hugo, 92200 Neuilly-sur-Seine, France

**Keywords:** Cardiology, Interventional cardiology

## Abstract

Patients’ prognostication around cardiac surgery is key to better assess risk–benefit balance. Preoperative brain natriuretic peptide (BNP) biomarker has been associated with mortality after cardiac surgery, but its added value with EuroScore 2 remains to be confirmed. In a prospective registry cohort of 4,980 patients undergoing cardiac surgery, the prognostic performance of EuroScore 2 and preoperative BNP was assessed regarding postoperative in-hospital mortality. Discrimination feature was evaluated using receiver-operator-characteristics analysis with area under curve (AUROC). Calibration feature was assessed using Hosmer–Lemeshow test. Multivariable analysis was performed to assess the association between covariates and in-hospital mortality. In-hospital mortality was 3.7%. The AUROC of EuroScore 2 was 0.82 (95% confidence interval (95%CI) 0.79–0.85, p < 0.0001). The AUROC of BNP was 0.66 (95%CI 0.62–0.70, p < 0.0001). The combined model with an AUROC of 0.67 (95%CI 0.63–0.71, p = 0.0001) did not yield better AUROC than EuroScore 2 alone (p < 0.0001 in disfavor of the combined model), nor BNP alone (p = 0.79). In multivariable analysis, EuroScore 2 remained independently associated with mortality (adj.OR of 1.12 (1.10–1.14), p < 0.0001), but BNP was not. Preoperative BNP was not an independent risk factor of postoperative mortality and did not add prognostic information, as compared to EuroScore 2 alone.

**Clinical trial registry** Registry for the Improvement of Postoperative OutcomeS in Cardiac and Thoracic surgEry (RIPOSTE) database (NCT03209674).

## Introduction

Accurate risk stratification in cardiac surgery is necessary to improve decision making prior to surgical and interventional treatment, patient information as to their prognosis and general^1^ care improvement.

The original European System for Cardiac Operative Risk Evaluation (EuroScore) was derived from a large international registry and enabled the estimation of postoperative mortality from clinical and biological preoperative variables^2^ EuroScore 2 was later developed to improve risk stratification in specific types of surgery such as aortic valve replacement (AVR) and improve overall calibration^3^.Ever since, EuroScore 2 has been largely accepted and is widely used. However, several concerns were raised, the score showing poor calibration in higher risk patients in whom it underestimated the risk^4^.

Apart from left ventricle ejection fraction (LVEF) and New York heart association (NYHA) functional class, EuroScore 2 does not capture heart failure severity, a known prognostic factor in cardiac surgery. Indeed, heart failure-related postoperative mortality is related to other factors than pump failure only, such as vasoplegia and systemic inflammatory response syndrome^3,4^. In a previous study, heart failure with preserved ejection fraction (HFpEF ) was shown to be an independent risk factor of mortality and post-operative shock^5^ in cardiac surgery. HFpEF was defined according to the 2016 European society of cardiology^6^ guidelines as an left ventricle ejection fraction ≥ 50%, symptomatic heart failure with New York heart association (NYHA) class 2 or greater, elevated brain natriuretic peptide (BNP) and relevant echocardiographic findings (left ventricle hypertrophy, left atrium enlargement, or diastolic filling anomaly).

Increased brain natriuretic peptide (BNP) plasma concentration has already been associated with worse outcomes in cardiac surgery^7–12^. However, although BNP is associated with mortality, interpretation must account for patients’ characteristics such as age, gender, morphology and renal function. While the predictive capabilities of BNP would not suffice to characterize patient-specific risk, this study tests the hypothesis that it could improve the accuracy of EuroScore 2.

The aim of this study was to evaluate the additional prognosis value of a model combining preoperative BNP and EuroScore 2 as compared to EuroScore 2 only, regarding in-hospital mortality.

## Results

### Demographics, types of procedures and outcomes

The scope of the study included 4,980 patients. Patients’ characteristics are presented in Table 1 and types of procedures in Table 2. Mean age was 68.9 years. Mean BMI was 26.7 kg/m^2^. The population was a standard population of heart surgery patients. 22% of the patients had a NYHA score above 3. 6% had diabetes, 13.2% had extra cardiac arteriopathy. 2.8% had active endocarditis. 81.2% had elective surgery.

**Table 1:** Baseline characteristics.

Demographics	Total n = 4,980	Alive (n = 4,797)	Dead (n = 183)	p-value
**Age (years)**	68.9 ± 11.0	68.7 ± 11	74.4 ± 9.7	0.003
**Female gender**	1,296 (26.0)	1,237 (25.8)	70 (38.2)	0.0001
**Weight (kg)**	77 ± 15.2	77.2 ± 15.2	73.6 ± 15.7	0.0001
**Height (cm)**	170 ± 9.12	170 ± 9	165 ± 9	0.0001
**BMI (kg/m**^**2**^)	26.7 ± 4.62	26.7 ± 4.6	26.6 ± 4.8	0.89
**EuroScore II**	3.21 ± 4.92	2.9 ± 4	11 ± 13.4	0.0001
**NYHA**				
II	954 (19.2)	933 (19.4)	21 (11.5)	0.0077
III	977 (19.6)	915 (19.1)	62 (33.9)	0.0001
IV	101 (2.0)	80 (1.7)	21 (11.5)	0.0001
**CCS 4**	62 (1.2)	54 (1.1)	8 (4.4)	0.0001
**Insulin-dependent diabetes mellitus**	293 (5.9)	283 (5.9)	10 (5.5)	0.8
**Extra-cardiac arteriopathy**	655 (13.2)	625 (13.0)	30 (16.4)	0.183
**Chronic obstructive pulmonary disease**	258 (5.2)	242 (5.0)	16 (8.7)	0.026
**Poor mobility**	72 (1.4)	66 (1.4)	6 (3.3)	0.03
**Renal dysfunction**				
On dialysis	58 (1.2)	48 (1.0)	10 (5.5)	0.0001
Creatinine clearance ≤ 50 mL/min	1,415 (28.4)	1,323 (27.6)	92 (50.3)	0.0001
Creatinine clearance 50–85 mL/min	2,434 (48.9)	2,371 (49.4)	63 (34.4)	0.0001
**Active endocarditis**	137 (2.8)	120 (2.5)	17 (9.3)	0.0001
**Critical preoperative state**	94 (1.9)	63 (1.3)	31 (16.9)	0.0001
**LV function**				
Moderate (31% to 50%)	773 (15.5)	734 (15.3)	39 (21.3)	0.028
Poor (21% to 30%)	127 (2.6)	113 (2.4)	14 (7.7)	0.0001
Very poor (below 20%)	16 (0.3)	12 (0.3)	4 (2.2)	0.0001
**Recent myocardial infarction**	232 (4.7)	212 (4.4)	20 (10.9)	0.0001
**PA systolic pressure**				
> 55 mmHg	273 (5.5)	248 (5.2)	28 (15.3)	0.0001
31–55 mmHg	1512 (30.4)	1,451 (30.2)	60 (32.8)	0.4672

Continuous variables are presented as mean ± standard deviation, categorical variables as number (percentage)

BMI: body-mass index; NYHA: New York Heart Association dyspnea grade; CCS: Canadian cardiovascular society angina pectoris grading; LV: left ventricle; PA: pulmonary artery.

**Table 2:** Types of procedure.

Type	
**Urgency of operation**	
Elective	4,067 (81.7)
Emergency	718 (14.4)
Urgent	189 (3.8)
Salvage	6 (0.01)
**Isolated CABG**	2,272 (45.6)
**Weight of procedure**	
1 non-CABG	1,580 (31.7)
2 procedures	934 (18.8)
3 or more procedures	86 (1.7)
**Valve procedures**	1942 (39.0)
**Aortic valve**	1644 (33.0)
**Mitral valve**	844 (16.9)
Repair	445 (8.9)
Replacement	391 (8.0)
**Tricuspid valve**	710 (14.3)
**Thoracic aortic surgery**	284 (5.7)
**Previous cardiac surgery**	227 (4.6)

Data are presented as number (percentage).

CABG: coronary artery bypass graft

The surgery was an isolated CABG in 45.6%, it was a valvular surgery in 39% of the surgeries.

EuroScore 2 had a median of 1.8 [IQR 1.0–3.42]. Preoperative BNP had a median of 481 ng/L [IQR 250–751].

Median length of stay in-hospital was 11 days [IQR 6–15]. In-hospital mortality rate was 3.7% (163 patients over 4,980 included). Comparing those who died to others, those who died were older (74.4 ± 9.7 vs. 68.7 ± 11, p-value = 0.003) and more often women (38.2 vs. 25.8%, p = 0.0001). Their EuroScore 2 was higher (6.0 [IQR 2.9–14.0] vs. 1.7 [IQR 1.0–3.2], p = 0.0001) with significant differences in NYHA scale, more chronic obstructive pulmonary disease, poor mobility, active endocarditis, critical preoperative state, and higher pulmonary pressure (see Table 1 for intergroup comparisons).

### Performance of EuroScore II

**Figure 1:**
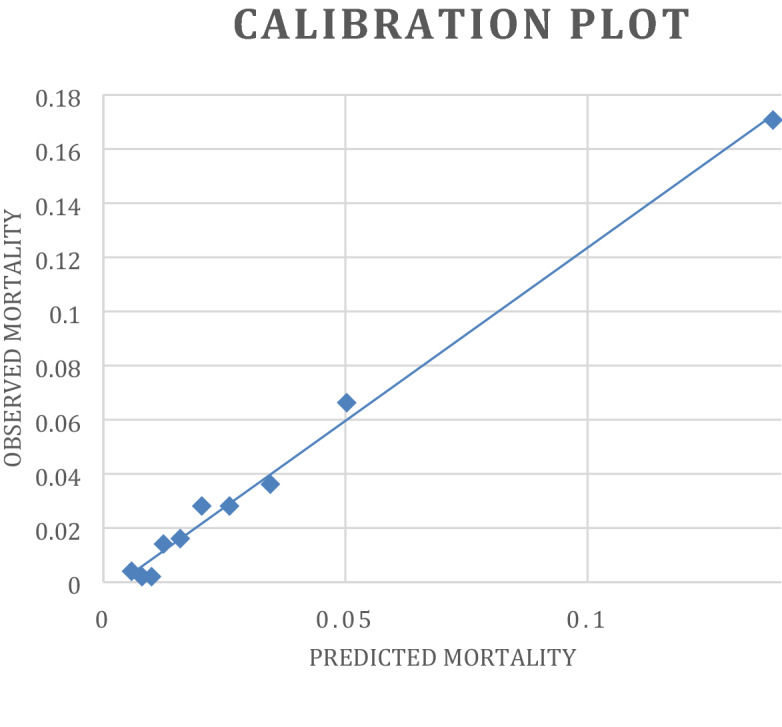
Calibration plot, comparison between observed mortality and mortality predicted by EuroScore 2. Differences between observed and predicted values are significant (Hosmer–Lemeshow test: χ^2^ = 49.94, p < 0.0001). i.e. for patients having a theoretical risk of 5%, observed mortality was 18% and for those predicted at 11%, had 30%; conversely, for patients predicted at 2%, observed mortality was null.

ROC analysis showed that EuroScore 2 had good discrimination with an AUROC of 0.82 (95%CI 0.79–0.85; p-value < 0.0001). Calibration measure with goodness-of-fit analysis showed significant differences between observed and predicted mortality (χ^2^ = 51; p-value < 0.00001). Differences were mostly observed in patients with EuroScore 2 below 2% where risk was overestimated, and above 5% where it was underestimated (see Fig. 1).

### Performance of preoperative BNP

ROC analysis showed that preoperative BNP adequately discriminated postoperative mortality with an AUROC of 0.66 (95%CI 0.62–0.70; p < 0.0001). Differences between observed and predicted values (calibration) are significant (Hosmer–Lemeshow test: χ^2^ = 49.94, p-value < 0.0001.

### Performance of a combined model

**Figure 2:**
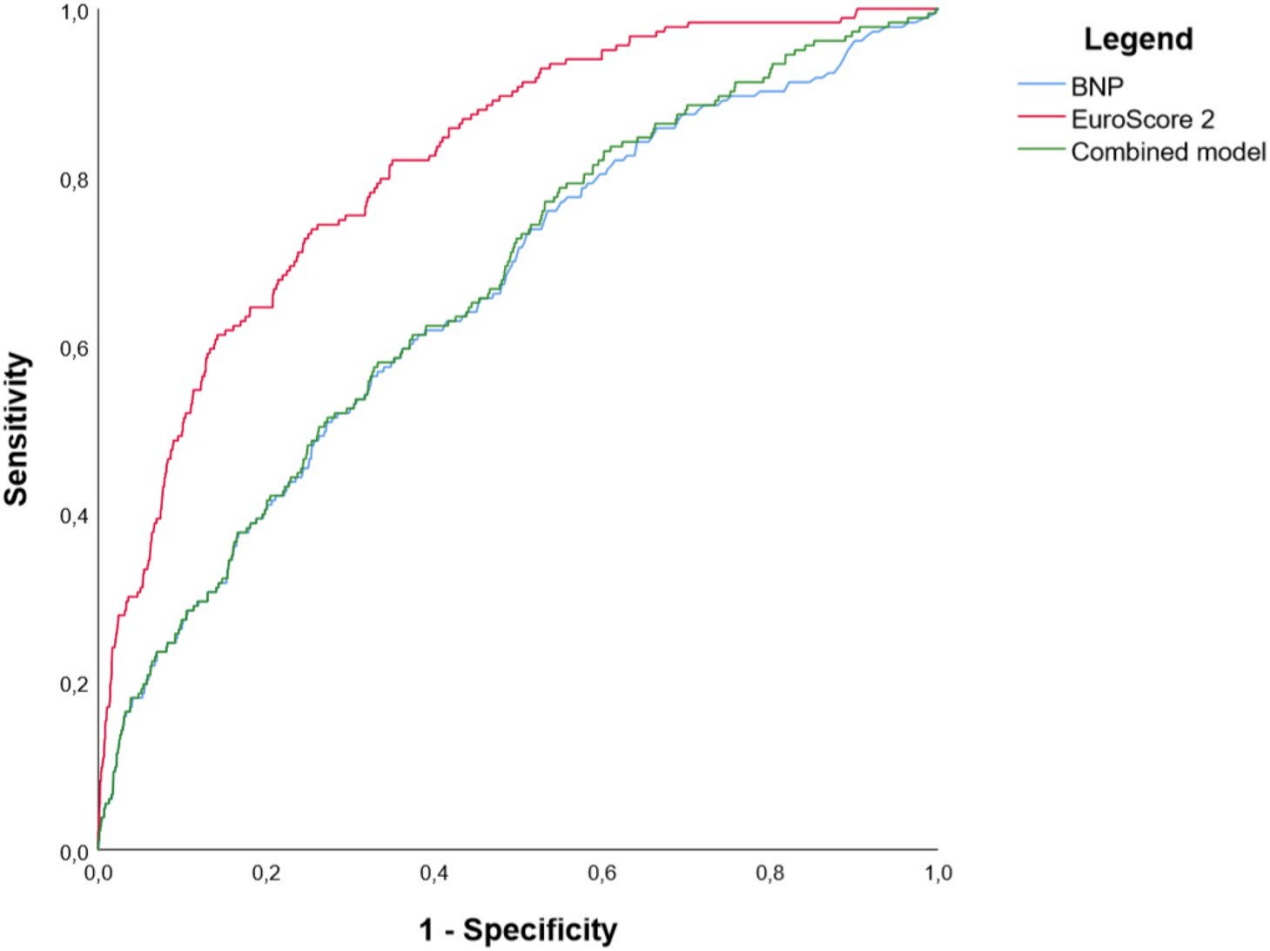
In-hospital mortality according to BNP, EuroScore 2 and a combined model. EuroScore 2 and preoperative BNP accurately discriminated in-hospital mortality with respective AUROC: 0.82 (95% CI 0.79–0.85), p < 0.0001 and 0.66 (95% CI 0.62–0.70), p-value = 0.0001. Combining EuroScore 2 and preoperative BNP yielded an AUROC of 0.67 (95% CI 0.63–0.71), p = 0.0001, however, it was not superior to neither EuroScore 2 alone nor BNP alone.”

ROC analysis showed that a combined model of EuroScore 2 and preoperative BNP adequately discriminated postoperative mortality with an AUROC of 0.67 (95%CI 0.63–0.71, p = 0.0001). The combined model did not show a better AUROC than EuroScore 2 alone, nor BNP (DeLong comparison test p < 0.0001 in disfavor of the combined model when compared to EuroScore 2 alone, and p = 0.79 when compared to BNP alone) (see Fig. 2).

### Association with in-hospital mortality

In univariate analysis, EuroScore 2 was associated with mortality with an unadjusted OR of 1.12 (1.10–1.14), p-value < 0.0001. Similarly, BNP was associated with mortality with an unadjusted OR of 1.06 (1.03–1.09), p-value < 0.001 (per 1,000 unit-increase).

In a multivariable analysis, EuroScore 2 remained independently associated with mortality with an adjusted OR of 1.12 (1.10–1.14), p-value < 0.0001. However, BNP was not associated with mortality anymore.

### Subgroup analysis with AUROC comparisons of EuroScore 2

We conducted a subgroup analysis of the prediction of in hospital mortality according to BNP and EuroScore 2 stratified on several factors (see Table 3).

**Table 3:** AUROC comparison, by subgroup.

	AUROC	Asymptotic 95% confidence interval (Lower bound–Upper bound)	p-value
**Discrimination of BNP**			
Age			
≤ 65 years	0.700	(0.599–0.800)	0.232204
> 65 years	0.630	(0.583–0.678)	
BMI			
≤ 25	0.627	(0.557–0.697)	0.26
> 25	0.679	(0.628–0.730)	
LVEF			
≤ 50	0.680	(0.602–0.758)	0.311
> 50	0.631	(0.581–0.681)	
EGFR			
≤ 60	0.576	(0.497–0.655)	0.08
> 60	0.664	(0.614–0.715)	
Emergency			
Elective	0.662	(0.616–0.708)	0.1177
Emergency	0.573	(0.470–0.676)	
Type of surgery			
CABG	0.693	(0.619–0.767)	0.18
Valve surgery	0.624	(0.573–0.674)	
**Discrimination of EuroScore 2**			
Age			
≤ 65 years	0.809	(0.732–0.886)	0.9
> 65 years	0.803	(0.769–0.838)	
BMI			
≤ 25	0.833	(0.782–0.885)	0.7
> 25	0.818	(0.783–0.854)	
LVEF			
≤ 50	0.811	(0.753–0.869)	0.98
> 50	0.812	(0.776–0.847)	
EGFR			
≤ 60	0.651	(0.571–0.730)	0.000058
> 60	0.845	(0.811–0.879)	
Emergency			
Elective	0.802	(0.767–0.837)	0.016
Emergency	0.671	(0.579–0.762)	
Type of surgery			
CABG	0.821	(0.763–0.879)	0.50
Valve surgery	0.791	(0.753–0.829)	

p-value represents intergroup comparison, using DeLong test (comparing two AUROCs).

In patients with a eGFR > 60 mL/min/m^2^, the AUROC of EuroScore 2 on mortality was higher than for those with eGRF less than 60 mL/min/m^2^ (respectively, 0.85 (0.81–0.88) vs. 0.65 (0.57–0.73), DeLong p-value < 0.0001). Similarly, in patients with an elective surgery the AUROC of EuroScore 2 was higher compared to the patients with emergency surgery (respectively, 0.80 (0.77–0.84) vs. 0.67 (0.58–0.76), DeLong p-value = 0.016).

Further sensitivity analyses were performed on subgroups, to assess association between BNP and in-hospital mortality, independently from EuroScore 2, using multivariable regression analysis. Overall, none were statistically significant, except in the subgroup of patients with eGFR > 60 mL/min/m^2^ (see Supplementary Material for details).

## Discussion

The study brought forward two main findings: (1) EuroScore 2 accuracy was validated regarding in-hospital mortality with an AUROC of 0.82 but undererstimated risk when above 10% and; (2) preoperative BNP was not independently associated with in-hospital mortality, regardless of EuroScore 2 in multivariable regression analysis.

The results concerning EuroScore 2 alone were consistent with previous publications: EuroScore 2 showed good discrimination with an AUROC of 0.81^1,13^ but limited calibration, trending towards underestimation of overall in-hospital mortality^2^. This limits the usage of this score as risk below 10% represents more than 90% of patients, notably for the choice between interventional procedures such as percutaneous valve replacement^14–17^ and conventional surgery. In patients with a low calculated risk score, additional risk stratification is necessary.

The purpose of this study was to see if an elevated pre-operative BNP could increase the stratification of in-hospital mortality for cardiac surgery, since it is a good predictor of mortality in conditions such as acute exacerbation of chronic obstructive pulmonary disease^18^, and patients with heart failure^19^.

BNP being secreted by cardiac ventricular myocytes in response to increased ventricular wall tension, it accounts for variations of heart filling conditions and has been reported more accurate than LVEF estimation regarding adverse outcomes, including mortality^2,20^. Indeed, BNP accounts for other types of heart failure than systolic dysfunction including right ventricle and diastolic dysfunction^21,22^. Furthermore, it provides a better understanding of the risk of endothelial and vascular dysfunction as well as the inflammatory respons^23,24^. As a matter of fact, BNP has been shown associated to postoperative extracorporeal-circulation-related systemic inflammation^4^.

The study showed that preoperative BNP discriminative performance was moderate with an AUROC of 0.66, albeit statistically significant, as previously documented^25^. However, in multivariable analysis, BNP was not independently associated with in-hospital mortality, and did not add to EuroScore 2, regarding risk evaluation. More importantly, when combined to EuroScore 2, BNP decreased discrimination assessed by AUROC, with a significantly lower AUROC. This may warrant caution when using this biomarker, on top of EuroScore 2, when assessing patients’ prognosis.

We previously conducted a subgroup analysis of patients with a normal LVEF^8^. According to the ESC definition, heart failure with preserved ejection fraction is defined by a normal LVEF and elevated BNP. As observed in the present study, an elevated BNP did not improve the prediction of mortality. Other markers such as diastolic dysfunction in echocardiography for example should be used^26^. Indeed, four elements may qualify for diastolic dysfunction: annular e′ velocity: septal e′ , lateral e′, average E/e′ ratio, left atrium volume index, and peak tricuspid regurgitation velocity.

Our assumption was that BNP may have added to the prognostication of cardiac surgery patients, by potentially identifying heart failure with preserved ejection fraction, however, this hypothesis was ultimately not proven in the present paper.

### Limitations

Although single-centred this study brought forward similar mortality rates and preoperative characteristics in our cohort to those previously reported in other cohorts, making external validation plausible but necessary.

BNP was routinely measured, instead of the more recent N-terminal pro-BNP (NT-proBNP), as the included patients were operated between 2012 and 2016. However, it has been documented that results found with BNP could be translated to NT-proBNP, regarding mortality and cardiac events^27,28^.

We acknowledge the collinearity between age^28^, eGFR^29^, LVEF^30^ and BNP. The sensitivity analysis that we conducted showed that even stratified on age, BMI, LVEF and type of surgery, BNP was not more associated with in-hospital mortality. Interestingly, in the subgroup of patients with eGFR above 60 mL/min/m^2^, BNP was an independent factor of mortality, albeit this result may require to be taken cautiously due to the type of analysis involved.

Finally, main outcome was in-hospital mortality, hence results may be harder to extrapolate to longer-term mortality, although, both EuroScore 2 and BNP were previously associated with longer-term mortality after cardiac surgery^14,31^.

## Methods

### Study population and study design

From the 1st of January 2012 to the 5th of July 2019, the study included all adult patients undergoing cardiac operations with cardiopulmonary bypass (CPB). Exclusion criteria were: age under 18 years and re-interventions during the same hospitalization. Informed consent was obtained from all subjects.

Data were collected prospectively: BNP and variables required for the computation of EuroScore 2 and in-hospital mortality. Data were anonymized as per national regulation and used with the approval of an institutional review board committee. All data are part of the Registry for the Improvement of Postoperative OutcomeS in Cardiac and Thoracic surgEry (RIPOSTE) database (NCT03209674). Patients’ opposition to the use of anonymized data by investigators was systematically sought (i.e. informed consent was obtained from all patients).

EuroScore 2 was computed as described in its original publication^32^. It included age, gender, New York Heart Association (NYHA) functional class, angina symptoms, insulin-dependent diabetes mellitus, extracardiac arteriopathy, chronic pulmonary dysfunction, neurological or musculoskeletal dysfunction severely affecting mobility, previous cardiac surgery, renal function with creatinine clearance, active endocarditis, critical preoperative state, left ventricle ejection fraction, recent myocardial infarction, pulmonary artery systolic pressure, procedure urgency and weight of the procedure.

Similarly to the EuroScore 2 study, main outcome was in-hospital mortality and was defined as death occurring in the same hospital where the operation took place, before discharge from the hospital^2^.

BNP was evaluated preoperatively in all patients, sampled in the 48 h preceding surgery. If several BNP levels were available, the most recent prior to surgery was used. BNP was quantified using immunoassay on Architect iSystem automatons (Abbott, Illinois, USA). Since BNP cut-off depends of studies and definitions^32,33^ we decided to use it as a continuous variable. All methods were carried out in accordance with relevant guidelines and regulations (Declaration of Helsinki).

### Data analysis

Descriptive analyses were conducted and expressed, for continuous variables as mean ± standard deviation or median [interquartile range, IQR] when appropriate; and for categorical variables as number of occurrences (percentage). Normality was assessed using a Shapiro–Wilk test.

Association between in-hospital mortality, EuroScore 2 and BNP was assessed using multivariable logistic regression.

Discrimination performance of the two models (EuroScore 2 alone and then the combined model) was assessed by building receiver operating characteristic (ROC) curves and by computing the area under curve (AUROC) with a 95% confidence interval [95%CI]. The AUROC were compared using Delong test^33^.

Calibration of EuroScore 2 was performed using the Hosmer–Lemeshow goodness-of-fit test, i.e. the same test used in the original validation paper of EuroScore 2^2^. Graphical representation was made by dividing EuroScore 2 into ranges of risk as described previously (< 1%, 1–5%, 5–10% and > 10%).

For ROC analysis, sample size required a minimum of 100 deaths to reach statistical significance^34,35^. With an expected mortality rate of 5%, based on our past activity and from other published registries, we required a minimum number of inclusions of 2,000 patients.

A multivariable analysis of mortality was performed with EuroScore 2 and BNP, using only these two variables as covariates. For sensitivity, subgroup analyses analysis were then conducted, based on specific subpopulations. They included age (under or above 65 years), BMI (under of above 25), LVEF (under and above 50%), and eGFR (under or above 60 mL/min/m^2^). Similarly, a subgroup analysis was conducted in patients with a low risk profile (EuroScore 2 under 2%) and a high risk profile (above 5%).

IBM v23.0 (IBM, Armonk, USA) was used for all analyses.

## Conclusion

In this cohort, while BNP adequately discriminated in-hospital mortality, it did not add prognostic value to EuroScore 2 regarding postoperative in-hospital mortality after cardiac surgery. EuroScore 2 underestimated mortality in patients with risk above 5%.

## Supplementary information

Supplementary file1 (DOCX 13 kb)

## Abbreviations

AUROC: Area under receiver operator characteristics’ curve
AVR: Aortic valve replacement
BMI: Body-mass index
BNP: Brain natriuretic peptide
CCS: Canadian cardiovascular society angina pectoris grading
EuroScore 2: European System for Cardiac Operative Risk Evaluation II
HFpEF: Heart failure with preserved ejection fraction
LVEF: Left ventricle ejection fraction
NYHA: New York Heart Association dyspnea grade
NT-proBNP: N-terminal pro-BNP
PA: Pulmonary artery
TR: Tricuspid regurgitation
