# Supplementary material for: Effect of BNP on risk assessment in cardiac surgery patients, in addition to EuroScore II
Source: Sci Rep. 2020 Jul 2;10:10865. doi: 10.1038/s41598-020-67607-0 (PMC7331739; doi:10.1038/s41598-020-67607-0)
Supplement: Supplementary file 1 — Supplementary file1 (DOCX 13 kb) [file 41598_2020_67607_MOESM1_ESM.docx]

Supplementary Material

For “*Effect of BNP on risk assessment in cardiac surgery patients, in addition to EuroScore II*” by Gaspard SUC, MD ; Philippe ESTAGNASIE, MD; Alain BRUSSET, MD; Niki PROCOPI, MD ; Pierre SQUARA, MD and Lee S. NGUYEN*, MD, PhDc

**Subgroup analysis with AUROC comparisons of EuroScore 2.**

The population was stratified according to its age (under of above 65 years), its BMI (under of above 25), its eGFR (under of above 60 mL/kg/min), its LVEF, the emergency status (elective or emergency), and the type of surgery (CABG or valve surgery). The AUROC were compared using Delong test. AUROC of BNP regarding in-hospital mortality did not differ when comparing theses subgroups. In those with an eGFR>60 mL/min/m2, AUROC tended to be higher than in those with eGFR<60 mL/min/m2 (respectively, AUROC (0.664 (95% CI 0.614 – 0.715) vs AUC : 0.576 (95% CI 0.497 – 0.655) ; DeLong p=0.08). Results are detailed in **Table 3** of main manuscript.

**Sensitivity analyses regarding association between BNP and in-hospital mortality (on top of EuroScore 2).**

Neither in patients aged more than 65 years-old nor below, was it significant. Similarly, BMI with a cut-off value of 25, LVEF with a cut-off value of 50%, elective surgery (compared to non-elective), isolated CABG (compared to combined surgery), low risk (EuroScore 2<2%) and high risk (EuroScore 2>5%) did not reveal any association between BNP and in-hospital mortality. Only in patients with eGFR above 60 mL/min/m2 was there a significant association between BNP and in-hospital mortality (per 100-unit increase, adj.HR=1.03 (95%CI=1.002-1.05), p=0.036).
